# Improvement of cancer subtype prediction by incorporating transcriptome expression data and heterogeneous biological networks

**DOI:** 10.1186/s12920-018-0435-x

**Published:** 2018-12-31

**Authors:** Yang Guo, Yang Qi, Zhanhuai Li, Xuequn Shang

**Affiliations:** School of Computer Science and Engineering, Northwestern Polytechnical University, Xi’an, 710072 China

**Keywords:** Cancer subtypes, Data integration, Biological network

## Abstract

**Background:**

Identification of cancer subtypes is of great importance to facilitate cancer diagnosis and therapy. A number of methods have been proposed to integrate multi-sources data to identify cancer subtypes in recent years. However, few of them consider the regulatory associations between genome features and the contribution weights of different data-views in data integration. It is widely accepted that the regulatory associations between features play important roles in cancer subtype studies. In addition, different data-views may have different contributions in data integration for cancer subtype prediction.

**Results:**

In this paper, we propose a method, CSPRV, to improve the cancer subtype prediction by incorporating multi-sources transcriptome expression data and heterogeneous biological networks. We extract multiple expression features of each genome element based on the regulatory associations in the heterogeneous biological networks and use a generalized matrix correlation method (*RV*_2_) to predict the similarities between samples in each view of expression data. We fuse the similarity information in multiple data-views according to different integration weights. Based on the integrated similarities between samples, we cluster samples into different subtype groups. Comprehensive experiments on TCGA cancer datasets demonstrate that the proposed method can identify more clinically meaningful cancer subtypes comparing with most existing methods.

**Conclusions:**

The consideration of regulatory associations between biological features and data-views contribution is important to improve the understanding of cancer subtypes. The proposed method provides an open framework to incorporate transcriptome expression data and biological regulation network to predict cancer subtypes.

**Electronic supplementary material:**

The online version of this article (10.1186/s12920-018-0435-x) contains supplementary material, which is available to authorized users.

## Background

Cancer disease is one of great danger to human health at present. Rather than being a single disease, a cancer type usually includes multiple subtypes in terms of different molecular pathogeneses and clinical features [1–5]. It is crucial to identify cancer subtypes to facilitate the precision of cancer diagnosis and therapy [6]. In recent years, the advance of high-throughput sequencing techniques produced large-scale sequencing data in diverse cancer types and made it easy to study cancer disease on genome molecular level [7, 8]. For example, The Cancer Genome Atlas (TCGA) pilot project [9] generated various types of cancer sequencing data on over 11,000 patient samples for over 34 cancer types [10]. The diverse genome-wide datasets allow researchers to improve cancer subtype prediction comprehensively using computational techniques. However, we still have limited subtype knowledge to human cancer diseases at present since the heterogeneity and complexity of cancers [5].

In recent years, many computational approaches were proposed to take the advantage of multiple types of cancer data to detect more clinically meaningful cancer subtypes [8, 11–14], such as iCluster [11, 13], CNMF (consensus non-negative matrix factorization) [13, 15], SNF (similarity network fusion) [8], WSNF [5] and ANF [16], etc. iCluster is a machine learning method to identify subtype clusters from multiple data sources by using EM algorithm, whereas feature selection is usually necessary for it works on high-dimensional data. CNMF is a modified non-negative matrix factorization method that identifies biological subtype patterns in multi-sources data by using non-negative matrix factorization approach. Nevertheless, it is time-consuming for it works on the high-dimensional biology data. SNF integrates multiple types of genomic data to identify cancer subtypes, while it uses iterative method to fuse similarity networks between samples, and the model is complex and hard to be interpreted in practice [16]. The recent HSNF [17] method enhanced the power of SNF, whereas the stability still need to be further improved. In addition, the feature importance provides useful information of features, and it hopes to improve the overall similarity estimation in data integration. WSNF incorporates the mRNA-TF-miRNA regulatory network information to predict the importance of each feature, and thus to identify cancer subtypes using SNF framework based on the weighted similarity information between samples. Although these integrative methods had been proven to be effective in subtype prediction, they did not consider the data-view weight in data integration, while different data-views may have different contributions to subtype prediction. To consider the contributions of different data-views, ANF [16] fuses multi-view affinity networks to identify cancer subtypes by incorporating multiple types of data. However, ANF did not consider the feature importance and the feature relationships in data integration. The heterogeneous biological regulatory network includes the relationships between features and it hopes to improve the subtype prediction by incorporating the network information in data integration, since different regulatory mechanisms may exist in different cancer subtypes.

In this paper, we propose CSPRV (Cancer Subtype Prediction using *RV*_2_ [18]) to improve the cancer subtype prediction by incorporating multi-sources expression data and heterogeneous biology regulatory networks. Given the expression data of genome elements, we first extract multiple expression features for each regulatory element based on the heterogeneous biological networks. Specifically, similar to [5], we consider gene (mRNA) and miRNA expression and mRNA-TF-miRNA regulatory network in this study (TF: transcription factor). We then reduce the extracted high-dimensional expression features to low-dimensional space and assemble them to construct an integrative feature matrix for each sample. Based on the extracted feature matrices of samples, we use a matrix correlation method, *RV*_2_ [18], to predict the similarities between samples in each expression data-view, and then fuse the similarity information in samples from all considering data-views according to different integration weights. Finally, we cluster patient samples into different cancer subtypes based on the predicted integrative similarity network between samples.

The main contributions of this study can be summarized as: (1) we consider more expression features for each focused genomic regulatory element based on the biological regulatory network in samples. (2) we use a generalized matrix correlation method to estimate the similarities between samples directly by considering the regulatory associations between features. (3) we take into account the contribution weights of different data-views in data integration. Comprehensive experiments based on TCGA BRCA and GBM datasets demonstrate that the proposed method is effective to identify more clinically meaningful cancer subtypes.

## Methods

**Fig. 1:**
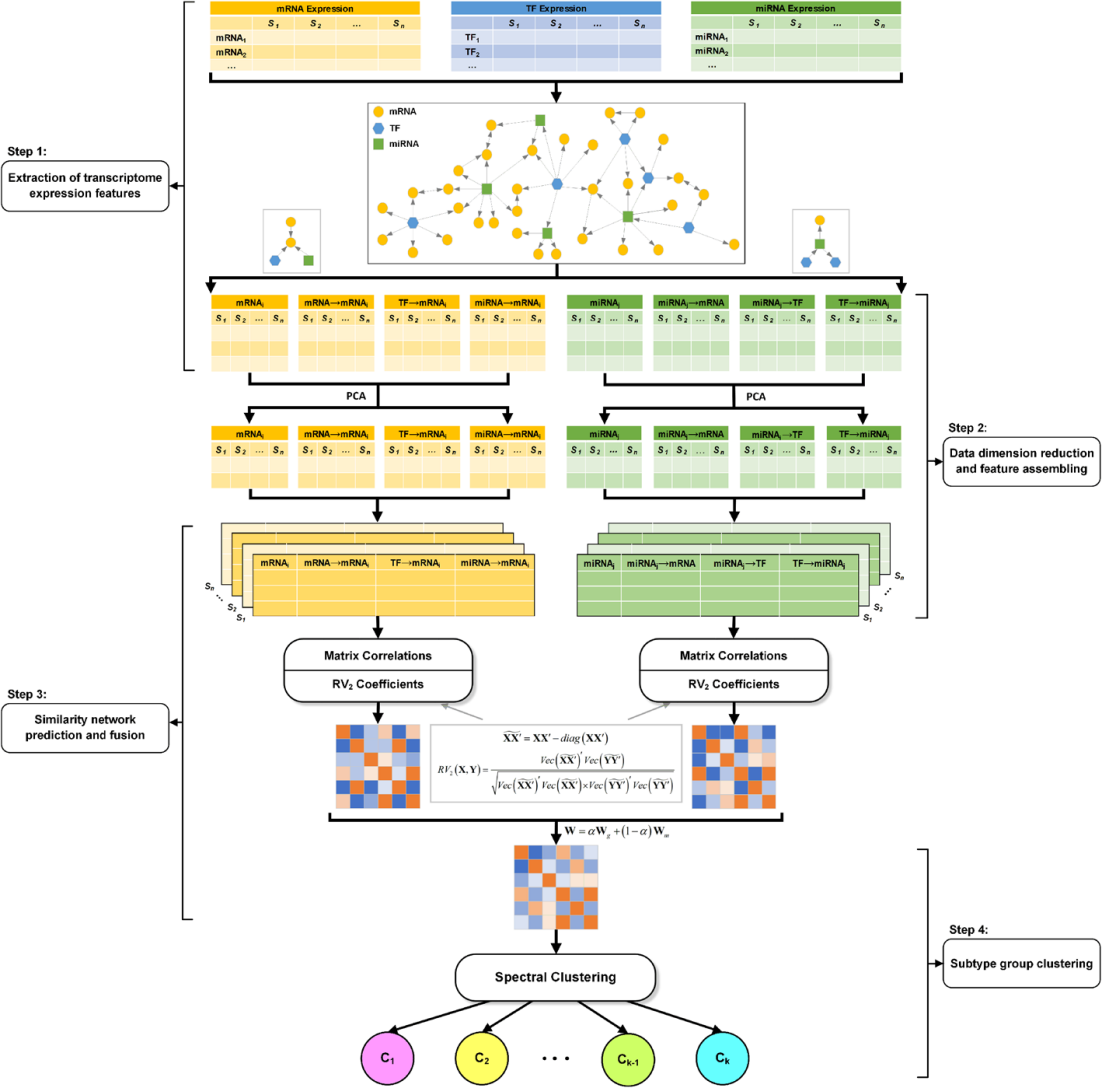
Overall procedure of the proposed integration framework. The mRNA-TF-miRNA heterogeneous regulatory network, gene (mRNA and TF) and miRNA expression data were considered in this study. *S*_*i*_ denotes sample *i*

We present a novel method that incorporates transcriptome profile data and mRNA-TF-miRNA regulatory network to predict cancer subtypes in cancer. The main idea is that we consider not only the transcriptome-wide expression of regulators but also the regulatory associations between them. For the transcriptome-wide expression data, we consider gene and miRNA expression data, respectively. Meanwhile, we integrate the mRNA-TF-miRNA regulatory network with the transcriptome expression data to consider associations between transcriptome regulatory elements. Figure 1 shows the overall procedure of our method in this paper. The details of each step are described as following.

### Step 1: Extraction of transcriptome expression features

**Fig. 2:**
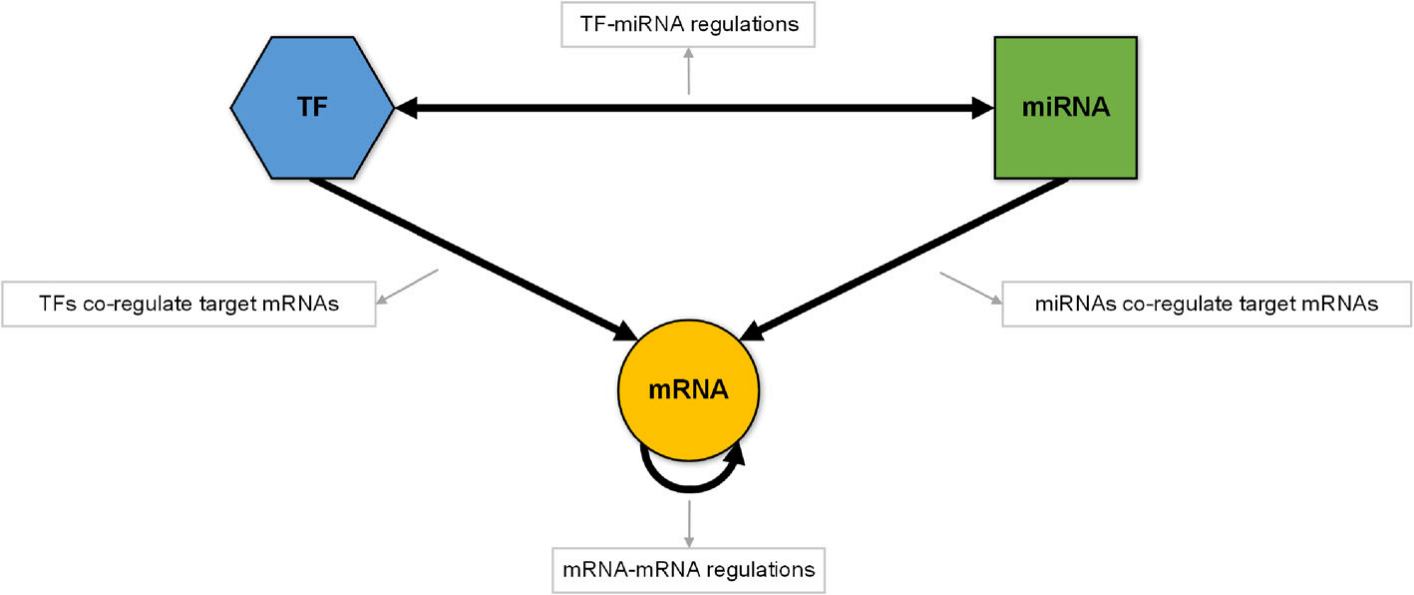
The basic biological regulation model between mRNA, TF (transcription factor) and miRNA

In this paper, we take into account two types of transcriptome expression data (gene: mRNA, if no specific states in following context, and miRNA expression) to integrate them with mRNA-TF-miRNA regulatory network for cancer subtype prediction. For each type of transcriptome expression features (gene or miRNA), we consider not only its expression, but also the expression features of its regulators or targets in the mRNA-TF-miRNA heterogeneous regulatory network. The intent to consider the features expression in network associations is that the expression of one considering genomic element is usually co-regulated by multiple regulators or links to multiple targets. There may exist situations that expression of the element have no much changes across samples, while the expression of its regulators or targets may have much difference across samples. These expression changes may have different regulatory patterns in different subtypes. The mRNA-TF-miRNA regulatory network includes the regulatory associations between mRNA ⇔mRNA, TF →mRNA, miRNA →mRNA and miRNA ⇔TF. Figure 2 shows the basic regulatory model between them in biological regulation process [5]. In the figure, a regulatory association is presented by a link, which the source corresponding to the regulator element and the end corresponding to the target.

**Fig. 3:**
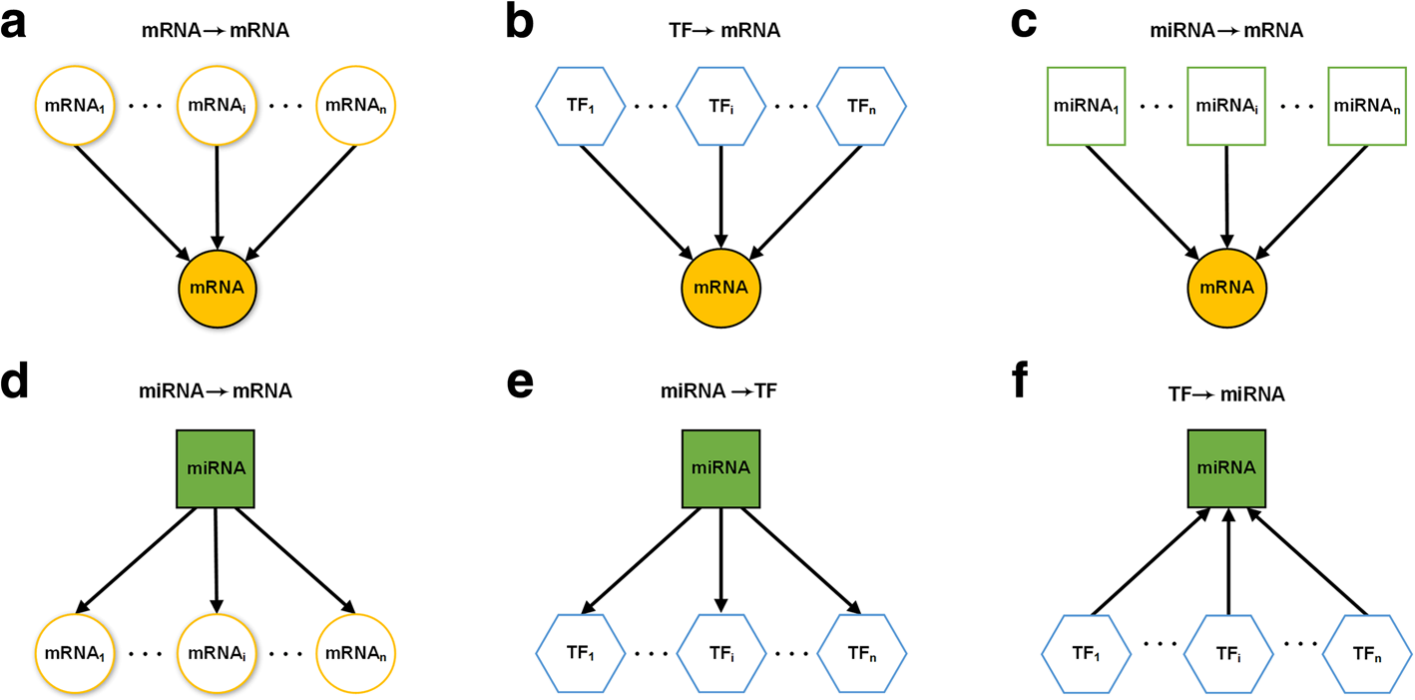
The schematics of the regulatory associations between different regulator elements in mRNA-TF-miRNA network. (**a**-**c**) Three types of regulatory associations centering on genes; (**d**-**f**) Three types of regulatory associations centering on miRNAs

Based on the basic biological regulation model, we extract multiple expression features for both genes and miRNAs by incorporating the regulatory association information in mRNA-TF-miRNA network. For the genes or miRNAs, we inspect the expression of them and their related transcription expression features from three kinds of regulatory associations in the network. As shown in Fig. 3, (a-c) shows schematics of the three kinds of regulatory associations centering on genes in the mRNA-TF-miRNA network; (d-f) shows schematics of the three kinds of regulatory associations centering on miRNAs in the mRNA-TF-miRNA network.

In detail, given a gene *g*_*k*_, four transcriptome expression features can be defined,

(1). Gene expression level.

1 $$ f_{0}= E\left(g_{k}\right)   $$

(2). Mean expression of its regulator mRNAs.

2 $$ f_{1}= f_{g_{k}}\left (mRNA\rightarrow mRNA_{g_{k}} \right)= \frac{\sum_{i=1}^{N}E\left (g_{i} \right)}{N}   $$

Where *E*(*g*_*i*_) is the gene expression of regulator gene *g*_*i*_ to *g*_*k*_, *N* is the number of the regulator genes of *g*_*k*_.

(3). Mean expression of its regulator TFs.

3 $$ f_{2}= f_{g_{k}}\left (TF\rightarrow mRNA_{g_{k}} \right)= \frac{\sum_{i=1}^{N}E\left (t_{i} \right)}{N}   $$

Where *E*(*t*_*i*_) is the expression of regulator TF *t*_*i*_ to *g*_*k*_, *N* is the number of the regulator TFs of *g*_*k*_.

(4). Mean expression of its regulator miRNAs.

4 $$ f_{3}= f_{g_{k}}\left (miRNA\rightarrow mRNA_{g_{k}} \right)= \frac{{\sum\nolimits}_{i=1}^{N}E\left (m_{i} \right)}{N}   $$

Where *E*(*m*_*i*_) is the expression of regulator miRNA *m*_*i*_ to *g*_*k*_, *N* is the number of the regulator miRNAs of *g*_*k*_.

Similarly, given a miRNA *m*_*k*_, four transcriptome expression features can be defined,

(1). miRNA expression level.

5 $$ f_{0}= E(m_{k})   $$

(2). Mean expression of its target mRNAs (genes).

6 $$ f_{1}= f_{m_{k}}\left (miRNA_{m_{k}}\rightarrow mRNA \right)= \frac{{\sum\nolimits}_{i=1}^{N}E\left (g_{i} \right)}{N}   $$

Where *E*(*g*_*i*_) is the expression of its target gene *g*_*i*_ of *m*_*k*_, *N* is the number of the target genes of *m*_*k*_.

(3). Mean expression of its target TFs.

7 $$ f_{2}= f_{m_{k}}\left (miRNA_{m_{k}}\rightarrow TF\right)= \frac{{\sum\nolimits}_{i=1}^{N}E\left (t_{i} \right)}{N}   $$

Where *E*(*t*_*i*_) is the expression of target TF *t*_*i*_ of *m*_*k*_, *N* is the number of the target TFs of *m*_*k*_.

(4). Mean expression of its regulator TFs.

8 $$ f_{3}= f_{m_{k}}\left (TF\rightarrow miRNA_{m_{k}} \right)= \frac{{\sum\nolimits}_{j=1}^{N}E\left (t_{j} \right)}{N}   $$

Where *E*(*t*_*j*_) is the expression of regulator TF *t*_*j*_ to *m*_*k*_, *N* is the number of the regulator TFs of *m*_*k*_.

As we stated above, for each gene or miRNA, four transcriptome expression features can be extracted by incorporating the corresponding regulatory associations in mRNA-TF-miRNA network. For each type of extracted transcriptome expression feature *i*, an expression matrix $E_{m\times n}^{\left (i \right)}$ can be used to denote the expression of all *m* genes/miRNAs in *n* patient samples in *i*. Therefore, four expression feature datasets for genes and four expression feature datasets for miRNAs can be extracted, respectively.

### Step 2: Data dimension reduction and feature assembling

The extracted transcriptome expression features are usually high-dimensional data. The high-dimensionality challenge may raise the extreme bias when using the data directly to predict the similarity information between samples. To ease the high-dimensionality challenge in this study, we use the principal component analysis (PCA) method [19, 20] to perform dimension reduction for each extracted expression feature matrix in both gene and miRNA data views. To obtain the same dimension for all gene/miRNA extracted expression features, and thus to facilitate the downstream similarity analyses, we reduce all gene/miRNA expression feature matrices to the largest components that ensure all four-expression datasets have least dimension redundancy and the explained variance rate > 0.9. Based on the reduction features, for each patient sample, we assemble all reduction gene/miRNA features from different expression feature matrices to compose two ensemble feature matrices in both gene and miRNA profile views (Fig. 1). The two ensemble feature matrices provide more comprehensive transcriptome expression features centering on genes and miRNAs respectively, which include not only expression information of both genes and miRNAs, but also the associations between them.

### Step 3: Similarity network prediction and fusion

Based on the processed feature matrices of samples, we calculate the correlation coefficient between feature matrices of samples. Specifically, given two feature matrices *X*^(*i*)^ of sample *i* and *Y*^(*j*)^ of sample *j*, we use the modified RV-coefficient (*RV*_2_) [18] method to calculate the correlation between *X*^(*i*)^ and *Y*^(*j*)^. The *RV*_2_ correlation coefficient between *X* and *Y* is defined as,

9 $$ {}RV_{2}\left (X,Y \right)\,=\, \frac{{Vec\left (\widetilde{X{X}'} \right)}'Vec\left (\widetilde{Y{Y}'} \right)}{\sqrt{Vec{\left (\widetilde{X{X}'} \right)}'Vec\left (\widetilde{X{X}'} \right)\times Vec{\left (\widetilde{Y{Y}'} \right)}'Vec\left (\widetilde{Y{Y}'} \right)}}   $$

The $\widetilde {X{X}'}= X{X}'-diag\left (X{X}' \right)$, where *diag*(*XX*^′^) is a matrix which only includes the diagonal elements of *XX*^′^ on its diagonal, and zero’s elsewhere. *X*^′^ is the transpose of *X*. *Vec*(*X*) is the symbol of the vectorized version of *X* [18]. The two most important advantages of *RV*_2_ method are: (1) it can measure the correlation between matrices directly, which contain more comprehensive features of samples; (2) it is robust to the matrix sizes and the correlation coefficient ranges from -1 to 1, which is similar to depicting the correlation between matrices as Pearson method.

Based on the ensemble feature matrices of samples, we predict expression correlations between samples in each data view. Then we use a scaled exponential kernel function to determine the similarity between samples by using a correlation-based distance measurement. Given two samples *X*_*i*_ and *X*_*j*_, the *RV*_2_ correlation coefficient is *ρ*(*X*_*i*_,*X*_*j*_), we defined the distance between *X*_*i*_ and *X*_*j*_ as,

10 $$ d\left(X_{i},X_{j} \right)= 1-\rho \left(X_{i},X_{j} \right)   $$

The similarity between *X*_*i*_ and *X*_*j*_ is similarly defined as [8],

11 $$ W\left (X_{i},X_{j} \right)= exp\left(-\frac{d^{2}\left (X_{i},X_{j} \right)}{\mu \varepsilon_{i,j}} \right)   $$

where *μ* is a hyper-parameter that can be defined empirically by users, the recommend range is [0.3, 0.8] in [8], and we set default value is 0.3 in this study. Here, the difference in our method is that we use different distance measurement in scaled similarity prediction. *ε*_*i,j*_ is a parameter that uses to eliminate the scaling problem, and it can be defined as [8],

12 $$ {}\varepsilon_{i,j}= \frac{mean\left(d\left({\vphantom{X_{j}}} X_{i},N_{i} \right)\right)+d\left(X_{i},X_{j} \right) + mean \left(d\left(X_{j},N_{j} \right) \right) }{3}   $$

where the *mean*(*d*(*X*_*i*_,*N*_*i*_)) is the average distance between *X*_*i*_ and all its neighbors.

We predict the similarities between samples centering on both genes and miRNAs, and fuse the similarity information according to the data weight parameters. For each pair of samples *X*_*i*_ and *X*_*j*_, the predicted similarity between them centering on gene and miRNA views are *W*_*g*_(*X*_*i*_,*X*_*j*_) and *W*_*m*_(*X*_*i*_,*X*_*j*_), respectively. Then the combined similarity between them is defined as,

13 $$ W_{c}\left (X_{i},X_{j} \right)= \alpha W_{g}\left (X_{i},X_{j} \right)+\left (1-\alpha \right)W_{m}\left (X_{i},X_{j} \right)   $$

where *α*∈[0,1].

### Step 4: Subtype group clustering

We use spectral clustering method to cluster patient samples into different subtype groups based on the integrative similarity information between samples. Concretely, suppose we define *m* subtype groups in *n* samples and a cluster partition matrix *Y*_*m*×*n*_=(*y*_1_,*y*_2_,⋯,*y*_*n*_) is used to indicate the labels of samples. For each sample *i*, *y*_*i*_ is the corresponding indicator vector, which *y*_*i*_∈{0,1}^*m*^. If *i* belongs to subgroup *k*, *y*_*i*_(*k*)=1, otherwise *y*_*i*_(*k*)=0. The spectral clustering method tries to identify the subgroup labels of samples according to solving the following optimal question [8],

14 $$ \underset{Q\in R^{n\times m}}{\min} Trace\left (Q^{T}L^{+}Q \right),s.t.Q^{T}Q=I   $$

where *Q*=*Y*(*Y*^*T*^*Y*)^−1/2^ is a scaled partition matrix, *L*^+^=*I*−*D*^−1/2^*WD*^−1/2^ is the normalized Laplacian matrix given the similarity matrix *W*. *D* is the diagonal matrix with diagonal elements being the sum of each row in *W*.

## Results

### Datasets

In this paper, we use the transcriptome datasets of two cancer types from TCGA, which are processed and provided in [5]. The two cancer types include breast invasive carcinoma (BRCA) with 587 samples and glioblastoma multiforme (GBM) with 276 samples. The BRCA datasets include gene (mRNA and TF) and miRNA expression data in all 587 samples. The gene and miRNA expression data are log2 transformation data and low expressed or uninformative genes and miRNAs are removed [5]. The gene and miRNA expression in GBM are microarray data, and the missing values are imputed and low expression or uninformative elements are removed [5]. The mRNA-TF-miRNA regulatory network data are obtained from [5], which are collected from several public databases, including ENCODE [21], TransmiR [22], TarBase [23] and STRING [24], etc. Since the network build on gene identities, to obtain more reliable network information, we map all gene identities to gene symbols and remove the genes with no mapped symbol from the heterogeneous network in each cancer data. To obtain consistent gene and miRNA list in both the gene/miRNA expression data and the network, we remove genes/miRNAs which are not in the network. Finally, in BRCA data, 11,396 genes (mRNAs), 1,302 TFs and 361 miRNAs are conserved in 587 samples; in GBM data, 9637 genes (mRNAs), 1059 TFs and 287 miRNAs are conserved in 276 samples. After collecting all expression data together, we use z-score standardized expression data across samples in our clustering pipeline. We also download the clinical data in each cancer type to evaluate the analysis results.

### Parameter selection

By integrating the gene/miRNA expression data with mRNA-TF-miRNA regulatory network together, we extracted more comprehensive expression features of each gene/miRNA. We used principal component analysis (PCA) method to reduce the high-dimensional feature data. In BRCA data, we finally conserved 268 and 124 components in gene and miRNA expression feature datasets, respectively (explained variance rate =0.92). In GBM data, we finally conserved 161 and 87 components in gene and miRNA expression feature datasets, respectively (explained variance rate =0.95). In fact, we tested it on the two cancer data and found the overall performance was not sensitive to the explained variance rate (Additional file 1: Tables S1 and S2). In our pipeline, to predict the subtype groups, one important hyper-parameter that needs to be tuned is the integrating data-view weight Eq. (13), which defines the similarity weight predicted on gene view in similarity integration. Different data-view weights may lead to different cancer subtype predictions. Before doing that, we set the default value *μ*=0.3 Eq. (11) in our study after testing the overall performance using different setting values (Additional file 1: Tables S3 and S4). To select the best *α* in each cancer data, we set *α* ranging from 0 to 1 with 0.1 differential at each trial and evaluated the subtype prediction performance on each parameter condition. Specifically, based on the clinical data in each cancer type, we performed the survival significance analyses for the identified cancer subtypes by using different integration parameters (*α*) and then evaluated the Cox log-rank test [25] *p*-value performance in survival time estimation. As studied in [5], five subtypes in breast invasive carcinoma data (BRCA) and three subtypes in glioblastoma multiforme cancer data (GBM) were defined. In this study, to be consistent with the previous study and fair to method comparison, we set the same cluster number in each cancer type data in clustering step as in [5].

**Fig. 4:**
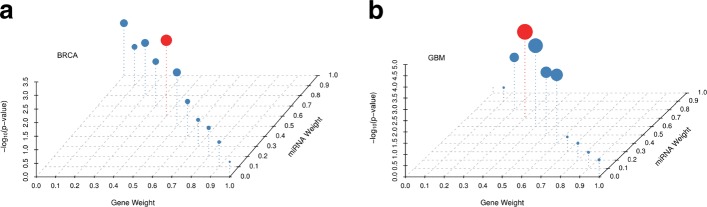
The Cox log-rank test *p*-value distribution in survival analyses of the identified subtypes using different integration weights in data integration. (**a**). *p*-value distribution in BRCA data; (**b**). *p*-value distribution in GBM data

Figure 4(a-b) show the Cox log-rank test [25] *p*-value performance of the identified cancer subtypes by using different similarity integration parameters in BRCA and GBM data. In each plot, the dot size is determined by the negative logarithm *p*-value in survival analyses. As shown in Fig. 4(a), in BRCA data, we obtain significant subtype prediction in survival analyses when *α* ranging from 0.0 to 0.5. Especially, when *α*=0.4, we obtain the most significant subtype estimation in survival analyses (*p*=1.60*e*−03). As shown in Fig. 4(b), in GBM data, we obtain significant subtype prediction in survival analyses when *α* ranging from 0.2 to 0.6. Especially, when *α*=0.3, we obtain the most significant subtype estimation in survival analyses (*p*=1.43*e*−04). Based on the performance in survival analyses of the identified cancer subtypes, we selected the best integration weight parameter to identify cancer subtypes in each cancer data in our downstream analyses. Importantly, we notice that there are different contributions of different data-views to subtype identification in the two cancer types. This may imply that there are different transcriptome regulatory mechanisms in different cancer types.

### Performance comparison with existing methods

We conducted cancer subtype prediction by using our proposed method on both BRCA and GBM data. To investigate the performance of the proposed method, we compared the performance of it with other seven existing integrative methods on the two cancer data. We investigated the Cox log-rank test *p*-value in clinical survival analyses for the identified cancer subtypes by using each method. It is reasonable that a good subtype prediction method should identify cancer subtypes with significant differential of survival time estimation. In comparison experiments, the number of subtypes in all two data were as same as in previous study [5] (five in BRCA and three in GBM). The seven reference integrative methods included CNMF [13, 15], iCluster [11], CC (consensus clustering) [13, 26], SNF [8], SNF-CC [13], ANF [16] and WSNF [5]. CC is a method can detect consensus clusters in multiple data sources. SNF-CC integrates the SNF and CC methods together to identify cancer subtypes from multiple datasets. Specifically, since the iCluster is high-computationally for high-dimensional data and feature selection is necessary in practice [13], in our experiments, we selected 10% of features if the number of features > 10,000 in one dataset, otherwise all features were used. In detail, in BRCA data, we selected the top 10% important features by the decreasing of variance. While, in GBM data, since all expression data were standardized data, all gene/miRNA had similar variance in samples. To test the overall performance of iCluster on these data, we run iCluster five times by randomly selecting 10% of features in the datasets which had feature number > 10,000, and used the median of *p*-values in survival analyses as the final reference. For other comparison methods, in all datasets, we set different parameters and used the best *p*-value in survival analyses as the final reference in the comparison. Table 1 shows the comparison performance of the identified subtypes in survival analysis by using different methods in two cancer datasets. As shown in Table 1, the proposed method predicts more significant cancer subtypes with survival difference in both BRCA (*p*=1.60*e*−03) and GBM (*p*=1.43*e*−04) datasets. Especially, considering the WSNF [5] method also incorporated the mRNA-TF-miRNA regulatory network to identify cancer subtypes, we find the proposed method consistently obtains more significant subtype prediction than WSNF in both BRCA and GBM datasets. This may because that we considered not only the transcriptome-wide expression of regulators but also the associations between them.

**Table 1:** Performance comparison of different integrative methods

Dataset	CNMF	iCluster	CC	SNF	SNF-CC	ANF	WSNF	CSPRV
BRCA	2.48e-02	9.70e-02	6.58e-02	5.64e-02	7.60e-02	2.98e-02	3.09e-02	1.60e-03
GBM	2.26e-01	1.72e-01	3.21e-01	2.53e-03	2.54e-03	8.72e-02	1.89e-03	1.43e-04

Cox log-rank test *p*-values of the identified subtypes in survival analyses were evaluated

### Cancer subtype prediction in breast cancer data

**Fig. 5:**
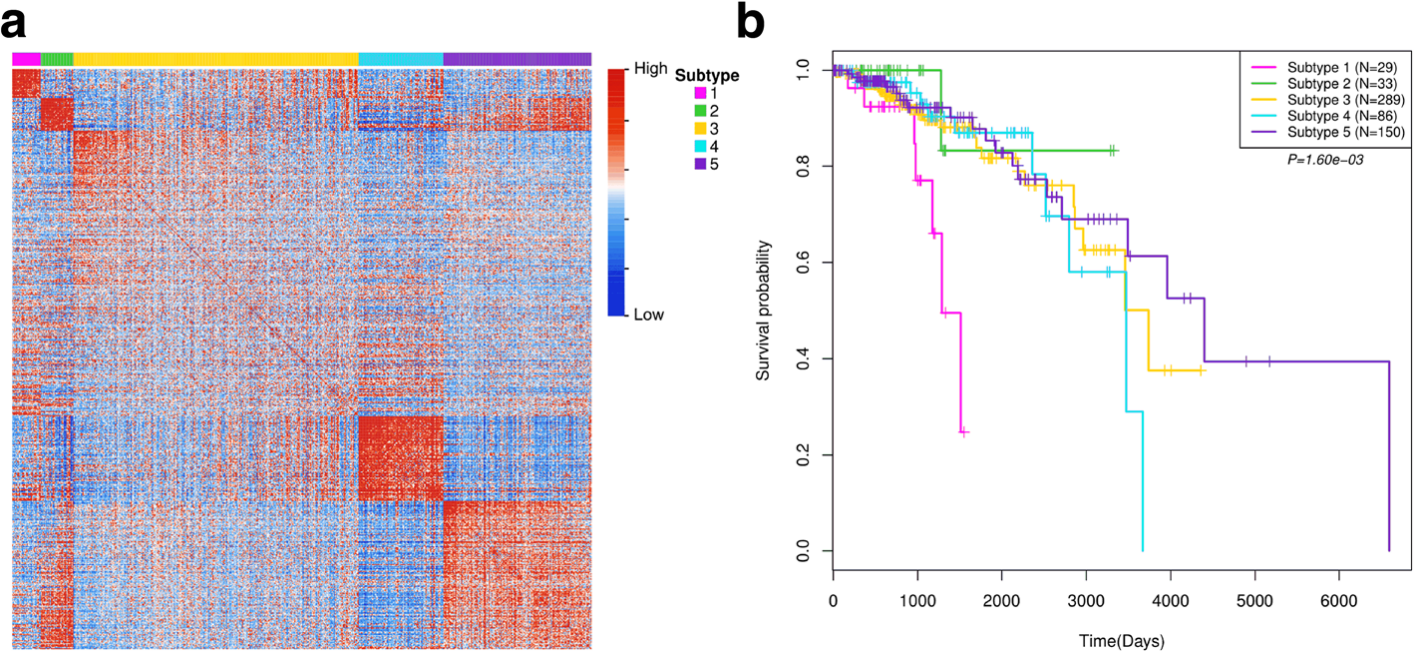
The identified subtypes in BRCA data. (**a**). Heatmap of the predicted integrative similarity network between samples (arranging samples by predicted subtype labels); (**b**). Kaplan-Meier survival probability curves of patients in the identified subtypes

We used the proposed method (CSPRV) to identify cancer subtypes in BRCA gene/miRNA expression datasets by incorporating mRNA-TF-miRNA regulatory network. Then, we evaluated the identified cancer subtypes based on various corresponding clinical data in breast cancer and analysed the differential of expression patterns of different transcriptome elements across subtypes. We identified five cancer subtype groups in breast cancer data. The five number of cancer subtypes in our study were defined as study in [5]. To investigate the consistence of the predicted cancer subtypes with the integrative similarity between samples, we generated the heatmap of the predicted integrative similarity network by arranging the samples according to the predicted subtype labels. As shown in Fig. 5(a), there are relatively clear block boundaries between different subtypes in the predicted similarity network between samples. This illustrates that the identified subtype groups are consistent with the predicted similarity information. The silhouette plots show that four-fifth clusters have positive silhouette score, while the largest one (cluster 3) have negative mean silhouette score (Additional file 1: Figure S1(a), visualization uses tool in [13]). This may be for the reason that the data noise of samples and the relatively weak discrimination of predicted similarity in cluster 3 samples. To consider this situation, we further investigated the significance of the clinical survival probability distributions of the identified subtypes. Kaplan-Meier survival analyses [27] were performed on the identified cancer subtypes. As shown in Fig. 5(b), there are significant differential of the survival probability estimations in different subtypes (*p*=1.60*e*−03). This illustrates that the CSPRV can detect clinically meaningful cancer subtypes in survival time estimation in breast cancer data, although the clustering of subtype cluster 3 is not as well as others. In addition, we also investigated the distributions of initial diagnosis ages and the survival time of the patients in each cancer subtype. Table 2 shows the average initial diagnosis age and survival time in each cancer subtype (ignored samples that have no corresponding information). We can see that there are not significantly different average diagnosis ages in the identified subtypes and most values are around 57 years old. This may illustrate that the breast cancer has higher risk of occurring in women around or over than 55 years old. In addition, we also notice that there are very different survival time in the identified subtypes. For example, in subtype cluster 1, the average survival time is only 920.0 days, while it is 2028.5 days in subtype 5. This indeed demonstrates that the identified subtypes provide significant clues in survival rate estimation.

**Table 2:** Average diagnosis ages and survival time of patients in the identified subtypes in BRCA data (Avg. survival time based on status=1 samples)

	Subtype 1	Subtype 2	Subtype 3	Subtype 4	Subtype 5
Avg. diagnosis age (years)	59.7	56.7	58.9	55.3	59.7
Avg. survival time (days)	920.0	1275.0	1309.1	1802.8	2028.5

**Fig. 6:**
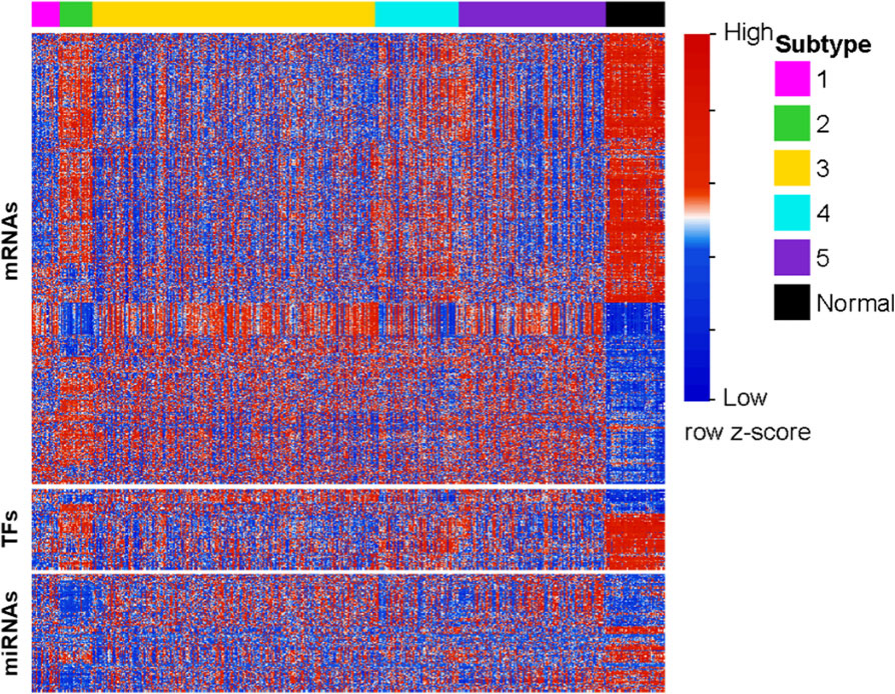
Heatmap of the differentially expressed mRNAs, TFs and miRNAs in the identified BRCA subtypes

To further recognize the transcription differential in the identified subtypes, we investigated the expression patterns of different transcriptome elements across the identified subtypes. Similar to the study in [5], we used 59 normal samples from TCGA as reference and detected the differentially expressed mRNAs, TFs and miRNAs across five identified subtypes. In detail, we used the voom [28] method in Limma [29] R package to detect the differential elements by comparing each subtype with normal group. We selected the differentially expressed (|*logFC*|>1.5; BH-adjusted-p < 0.01) genes (920 mRNAs and 81 TFs) and miRNAs (119 miRNAs) in all subtype groups (excepted common elements in all subtypes), and investigated the expression patterns of them in our analyses. As shown in Fig. 6, there are relatively clear different expression patterns in subtypes comparing with the normal group, especially in the mRNA and miRNA datasets. We also checked the overlaps of the differentially expressed mRNAs/miRNAs in five cancer subtypes using the normal group as reference (Additional file 1: Figure S2(a-b)). We notice that there are not only some common changes in all subtypes, but also subtype-specific changes in gene and miRNA expression data, respectively.

**Fig. 7:**
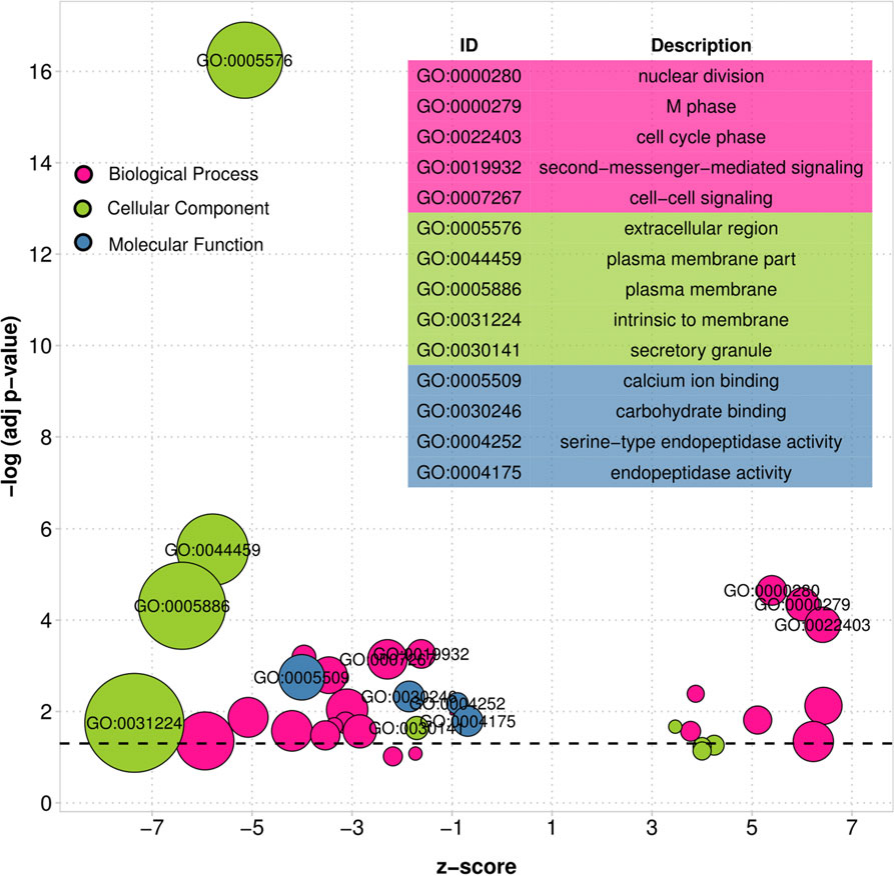
Gene Ontology enrichment analysis of the differentially expressed genes (mRNAs) in BRCA subtypes. z-score provides hint of a term related to the up-regulated (positive) or down-regulated (negative) genes [31]. The dash line is corresponding to adjusted *p*-value=0.05

Moreover, to check the biological functions of the genes that expression alternatively changes across subtypes (excepted common differential genes), we conducted Gene Ontology enrichment analysis on these genes using tools in [30, 31]. Figure 7 shows the situation of the enrichment ontology terms of those cancer related genes (only shown the terms with BH-adjusted-p < 0.1). As shown in Fig. 7, we can see that the most significant enrichment terms are related to the cell division, cell cycle phase, cell-to-cell signaling, etc. in biological process. Some of these enrichment functions are actually related to the breast cancer development, such as cell division, cell signal transition, etc. This may reveal that the different regulation patterns in biology cells in cancer subtypes. We also conducted enrichment analysis on those cancer-related miRNAs by using the miEEA [32] tool in breast cancer. Table 3 shows the top-5 most significant related terms. As shown in Table 3, these miRNAs are enriched on the cancer diseases. This indeed demonstrates that the abnormal expression of miRNA is one of important factors to affect the cancer occurrence and thus raise different cancer subtype attributions.

**Table 3:** Disease, pathway and gene ontology enrichment analyses on the differentially expressed miRNAs in BRCA subtypes

Disease	Adj-*p*-value
Neoplasms	1.31e-06
Carcinoma	8.93e-05
Adenocarcinoma	1.50e-03
Ovarian Neoplasms	1.97e-03
Breast Neoplasms	4.15e-03
Pathway	Adj-*p*-value
P00011 Blood coagulation	9.76e-04
WP272 Blood Clotting Cascade	6.86e-03
P00045 Notch signaling pathway	9.88e-03
WP129 Matrix Metalloproteinases	9.88e-03
WP138 Androgen receptor signaling pathway	9.88e-03
Gene Ontology	Adj-*p*-value
GO0005515 protein binding	2.90e-03
GO0035019 somatic stem cell maintenance	2.90e-03
GO0042246 tissue regeneration	2.90e-03
GO0042742 defense response to bacterium	2.90e-03
GO0030177 positive regulation of wnt receptor signaling pathway	3.95e-03

Top-5 significant enrichment terms are shown in each category

### Cancer subtype prediction in glioblastoma multiforme data

**Fig. 8:**
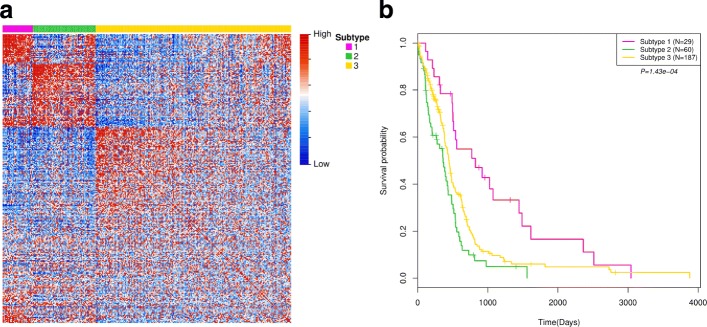
The identified subtypes in GBM data. (**a**). Heatmap of the predicted integrative similarity network between samples (arranging samples by predicted subtype labels); (**b**). Kaplan-Meier survival probability curves of patients in the identified subtypes

To further demonstrate the effectiveness of the proposed method in cancer subtype identification, we used it to identify cancer subtypes in glioblastoma multiforme cancer data (GBM). Similar to the study in [5], we defined three subtype clusters and used the best *α* parameter to predict subtypes in GBM data (Fig. 4(b)). Figure 8(a) shows heatmap of the predicted integrative similarity network by arranging the samples according to the predicted subtype labels. There are clear block boundary between subtypes. This illustrates that the predicted subtypes are consistent with the estimated integrative similarity information between samples. The silhouette plots of the subtype clusters show all of them have positive global silhouette scores and the overall score is 0.12 (Additional file 1: Figure S1(b), visualization uses tool in [13]). This illustrates that most samples have well cluster prediction in GBM data. Figure 8(b) shows the Kaplan-Meier survival probability curves of the identified cancer subtypes in clinical survival analyses. We find that there are very different survival probability estimations in the identified subtypes (*p*=1.43e-04). This indeed illustrates that CSPRV can detect clinically meaningful cancer subtypes in survival time estimation in GBM data. Similarly, we also investigated the distribution of the initial diagnosis ages and the survival time of the patients in each cancer subtype and found that they were significantly different in cancer subtypes (Table 4, ignored samples that have no corresponding information). As shown in Table 4, in subtype cluster 1, the average initial diagnosis age is 51.1 years and the average survival time is 935.2 days; however, in the subtype cluster 2, the average initial diagnosis age is 61.6 years (older 10 years than in cluster 1) and the average survival time is only 346.3 days. This may illustrate that there are very different age-related risks and survival rates in the glioblastoma multiforme disease in different cancer subtypes. To further study the effect of age factor to the survival rate in different cancer subtypes, we simply divided the patient samples into two groups by using 60-years cutoff, which corresponding to non-old (age <=60) and old (age > 60) groups (peak in age distribution in Additional file 1: Figure S3).

**Table 4:** Average diagnosis ages and survival time of patients in the identified subtypes in GBM data (Avg. survival time based on status=1 samples)

	Subtype 1	Subtype 2	Subtype 3
Avg. diagnosis age (years)	51.1	61.6	58.1
Avg. survival time (days)	935.2	346.3	490.2

**Fig. 9:**
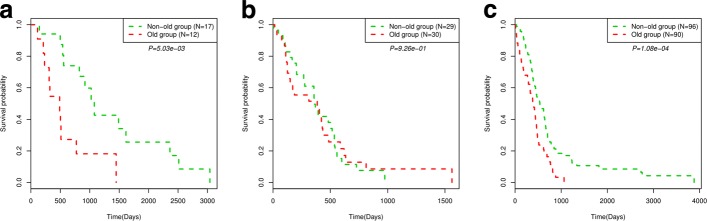
Kaplan-Meier survival probability curves of patients in two age groups in the identified subtype clusters in GBM data (ignored samples that have no age information). (**a**-**c**)The identified subtype 1, 2 and 3, respectively

We performed survival analyses on the two age groups in each subtype. As shown in Fig. 9(a-c), there are significant survival differential between the two age groups in subtype cluster 1 and 3 (Cox log-rank test *p*-value 5.03e-03 and 1.08e-04, respectively); while in the subtype cluster 2, there is not significant differential of the survival probability between two age groups. This illustrates that age factor may have different effects to the survival anticipate in different subtypes.

## Discussion and conclusions

Identification of cancer subtypes is of great importance to cancer diagnosis and therapy. Many approaches were proposed to integrate multi-sources data to identify cancer subtypes in recent years, such as iCluster [11], SNF [8], WSNF [5] and ANF [16], etc. SNF, on behalf of the recent integration methods, is an effective and efficient method that fuses multi-sources data according to similarity networks between samples. It can discover cancer subtypes with different survival patterns by integrating multi-sources data. In view of SNF does not consider the importance of features in similarity fusion, WSNF predicts the importance of features by incorporating mRNA-TF-miRNA regulatory network, and then predict the subtypes based on the integrative similarity information between samples by using SNF framework. It is proved that the heterogeneous regulatory network information is useful to assist similarity estimation between samples. In this study, we consider not only the regulatory associations between features in mRNA-TF-miRNA regulatory network, but also the weights of different data-views. Comparing with WSNF predicts the feature weights directly, CSPRV extracts multiple expression features for each genomic feature based on the heterogeneous network and uses *RV*_2_ matrix correlation method to predict the similarity information between samples. In fact, the extracted features also include the feature importance information in high-dimensionality. In addition, CSPRV also considers the weights of different data-views in data integration according to manually defining the weight parameters in algorithm. This strategy is more robust to make the method to work on different cancer data.

Although the proposed method detected more significant subtypes in survival estimation on the two cancer datasets, the robustness of the method still need to be further improved in future. For examples, as the visualization of the integrative similarity in Fig. 5(a), the largest subtype (cluster 3) is not clear as well as other four clusters (silhouette score is -0.04) in BRCA data. This illustrates that the discrimination of the predicted similarities between samples are not enough in some extent, especially in the samples with large data noise. Since we predict the similarity information based on the correlation version distance, good data features and data noise processing are important to similarity prediction. The performance of the proposed method hopes to be improved by considering more data information. On the one hand, in this study, we currently based on the datasets in [5], and the network only includes mRNA, TF and miRNA features. More accurate and complete heterogeneous networks hope to provide more comprehensive transcriptome expression information, thus to improve the performance on cancer subtype prediction. On the other hand, to handle the data high-dimensionality challenge, we used the PCA method to perform dimension reduction in feature integration at present, which is a linear model in feature embedding. More robust and complex feature-embedding methods may hope to be used to improve the quality of the learned features. We plan to extend and improve the method in future.

In conclusion, we proposed a new model, CSPRV, to integrate multiple types of transcriptome expression data and heterogeneous biological network to identify cancer subtypes. Tests on TCGA BRCA and GBM datasets demonstrated that the proposed method obtained more significant subtypes, which shown different survival patterns. We hope it could be a useful approach to facilitate the cancer disease analyses in future.

## Additional file

Additional file 1 Figures S1-S3 and Tables S1-S4. (PDF 526 kb)
