# Supplementary material for: Improvement of cancer subtype prediction by incorporating transcriptome expression data and heterogeneous biological networks
Source: BMC Med Genomics. 2018 Dec 31;11(Suppl 6):119. doi: 10.1186/s12920-018-0435-x (PMC6311915; doi:10.1186/s12920-018-0435-x)
Supplement: Supplementary file 1 — Figures S1-S3 and Tables S1-S4. (PDF 526 kb) [file 12920_2018_435_MOESM1_ESM.pdf]

Additional Figures & Tables

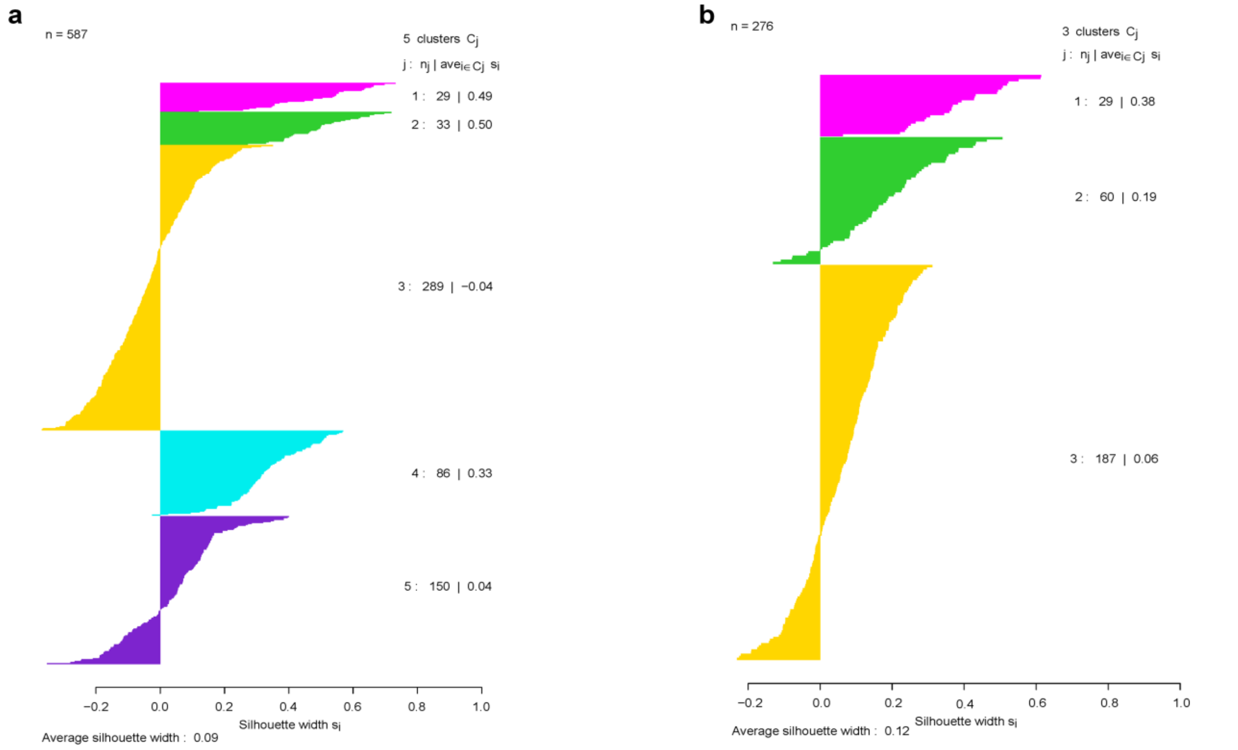

Figure S1. Silhouette plots of the identified subtype clusters in clustering. (a) Silhouette plots of subtype clusters in BRCA data. (b) Silhouette plots of subtype clusters in GBM data.

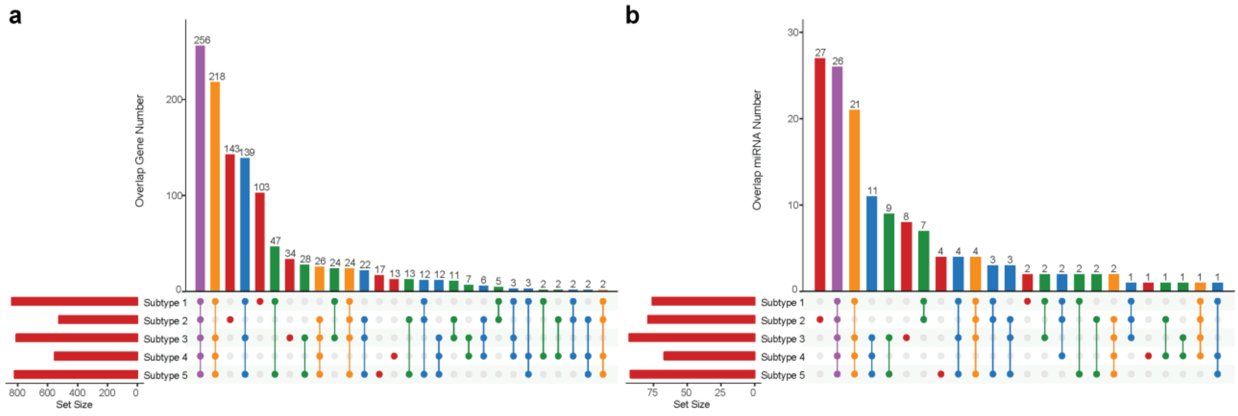

Figure S2. Overlaps of the differentially expressed mRNAs and miRNAs in the identified BRCA subtypes. (a) Overlaps of differentially expressed mRNAs in BRCA subtypes; (b) Overlaps of differentially expressed miRNAs in BRCA subtypes.

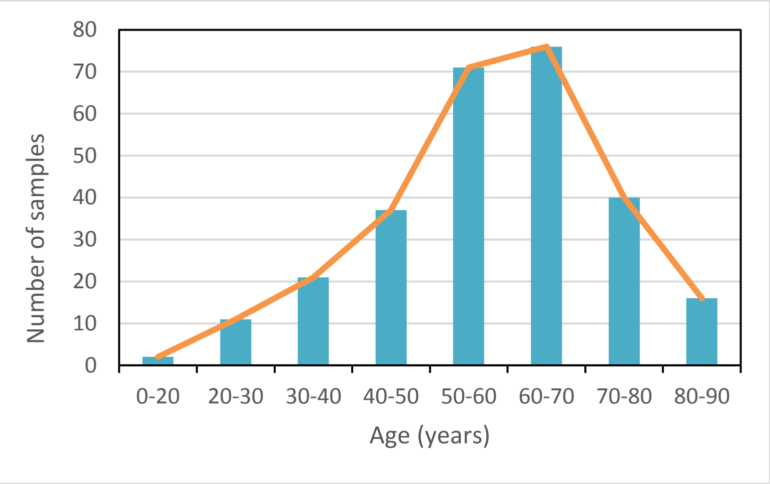

Figure S3. Age distribution of samples in GBM data.

Table S1. Cox log rank test p-values of the identified subtypes by using different dimension reduction and data integration parameters in BRCA data.

**PCA explained variance rate**	***r*** **=0.85**	***r*** **=0.90**	***r*** **=0.92**	***r*** **=0.95**
**Integration α =0.3**	7.93e-03	1.92e-02	2.30e-02	2.36e-02
**α =0.4**	1.06e-03	1.91e-03	1.60e-03	7.12e-03
**α =0.5**	2.10e-02	1.79e-02	1.03e-02	9.26e-03
**α =0.6**	3.99e-02	9.43e-02	5.28e-02	7.19e-02

Table S2. Cox log rank test p-values of the identified subtypes by using different dimension reduction and data integration parameters in GBM data.

**PCA explained variance rate**	***r*** **=0.85**	***r*** **=0.90**	***r*** **=0.95**
**Integration α =0.3**	5.79e-04	3.56e-04	1.43e-04
**α =0.4**	4.00e-04	2.86e-04	2.51e-04
**α =0.5**	1.00e-03	1.22e-03	1.62e-03
**α =0.6**	1.57e-03	1.11e-03	8.95e-04

Table S3. Cox log rank test p-values of the identified subtypes by using different data integration and scaling hyper-parameters in BRCA data.

Parameters	α =0.3	α =0.4	α =0.5	α =0.6
μ =0.3	2.30e-02	1.60e-03	1.03e-02	5.28e-02
μ =0.4	6.18e-01	6.92e-01	4.95e-02	5.10e-02
μ =0.5	2.72e-01	7.06e-01	7.19e-02	2.52e-01
μ =0.6	2.84e-01	4.11e-01	3.62e-01	2.07e-01
μ =0.7	3.83e-01	2.00e-01	1.30e-01	2.06e-01
μ =0.8	3.83e-01	1.94e-01	1.41e-01	2.62e-01

Table S4. Cox log rank test p-values of the identified subtypes by using different data integration and scaling hyper-parameters in GBM data.

Parameters	α =0.3	α =0.4	α =0.5	α =0.6
μ =0.3	1.43e-04	2.51e-04	1.62e-03	8.95e-04
μ =0.4	1.73e-02	3.93e-03	1.87e-03	1.41e-01
μ =0.5	2.18e-02	3.24e-03	2.00e-03	1.76e-01
μ =0.6	3.39e-02	1.63e-02	1.83e-03	1.78e-01
μ =0.7	7.48e-02	1.00e-01	4.47e-03	1.78e-01
μ =0.8	7.48e-02	8.50e-02	4.15e-03	1.78e-01
